# New York Odontological Society

**Published:** 1891-10

**Authors:** 

**Affiliations:** New York Odontological Society


### Reports of Society Meetings.

### NEW YORK ODONTOLOGICAL SOCIETY.

A regular meeting of the New York Odontological Society was held on Tuesday evening, June 16, 1891, at the New York Academy of Medicine, No. 17 West Forty-third Street, New York City.

The President, Dr. William H. Dwinelle, in the chair.

### REPORT OF COMMITTEE ON THE DEATH OF DR. EDWARD MAYNARD.

Your committee appointed to draft resolutions in regard to the death of Dr. Edward Maynard beg leave to report the following:

Since our last meeting, following up the death record which has been so appalling of late, an honored member of our profession, a distinguished citizen and man of the world, has taken his departure from our midst. We refer to Dr. Edward Maynard, who died in Washington, May 4, 1891.

Dr. Maynard's relation to our profession was a peculiar one. He may be considered, as far as this country is concerned, to be one of its founders. His early associates were Drs. Hayden Harris, the Parmlys, Solyman Brown, Elisha Townsend, and the noble army of worthies of that day. He was one of the editors of the *American Journal of Dental Science* in 1844, and occupied the chair of Theory and Practice in the Baltimore College of Dental Surgery in 1857.

In consideration of his contributions to science and art he received honorary degrees from various institutions abroad, and diplomas of merit from our most distinguished seats of learning. His reputation as an inventor extends throughout the civilized world.

He was appointed as a cadet at West Point by De Witt Clinton, but resigned a year after on account of ill health. He then gave his attention to civil engineering, anatomy, architecture, drawing, and such mechanical employments as would best educate his hand for the profession of dentistry, which he finally adopted in 1835.

By invitation of the Hon. Secretary of War, Dr. Maynard was one of the gentlemen who attended the examination of the cadets of the Military Academy at West Point, in June, 1863, and was nearly successful in his endeavor to have a Dental Corps attached to the army and navy, justly considering the care of the teeth of officers and soldiers of paramount importance.

He was a many-sided man, and was an expert in regard to whatever passed through his hands, as attested by the varied instruments that came from his inventive mind, whether for the dentist, the surgeon, or for the world's service at large, as exemplified by his rifle, which has made him famous even beyond the bounds of civilization.

He embraced many departments of art, and made them all tributary to our profession. He gave an air of finish, of grace and beauty, to all his productions, and put the stamp of excellence upon everything that passed through his hands. Honors so abundantly bestowed upon him he laid as trophies at our feet, and was proud to do so.

He was an expert in wood-engraving, many specimens of which are still in existence. In wood-carving he showed great ability. Some of his earlier efforts in modelling in clay gave great promise in this direction. In drawing and color he was particularly happy. No architectural drawings could exceed his in correctness, in detail and finish; they reminded one of the best efforts of the most accomplished experts.

He was a member of the celebrated Sketch Club at Washington, where he, with others, spent evenings sketching from the nude.

For many years, like Washington, he was a surveyor, and some of our most important public works attest his skill and direction.

He was employed by our government to make the first experiments, in conjunction with the authorities in Belgium, in regard to the manufacture of Damascus steel.

All of his models, his inventions in guns, his medals and decorations, are deposited in the National Museum at Washington, in cases especially set apart for them, for the purpose of showing everything in the line of fire-arms of his invention from the earliest to his latest.

We have mentioned these things thus in detail to show that reflectively they redound to the honor and glory of our profession, to which he was so much attached.

Modest, retiring almost to diffidence, delicate and refined even to the graces of womanhood, Dr. Maynard presents to us a character for our contemplation such as we cannot expect to meet again. He has placed us under such obligations to him that no time can obliterate, and we desire to put on record our sense of that obligation and of our great loss.

Dr. Maynard has two sons now living in this city,—George W. Maynard, an artist of eminence, and John D. Maynard, who follows the profession of his distinguished father.

*Resolved*, That a copy of these resolutions be published in the proceedings of this Society, and that a copy of them be sent to the surviving family of Dr. Maynard.

William H. Dwinelle,
S. G. Perry,
C. E. Francis,
Benjamin Lord,
*Committee.*

*Dr. Remington.*—I move that the report just read be spread in full upon a memorial page of our minute-book, and that a copy of the report be sent to the family of the deceased.

Motion seconded and carried.

*The President.*—Gentlemen, I have the pleasure of introducing to you as our essayist for this evening, Horatio C. Meriam, D.M.D., of Salem, Mass., who will now address us.

(For Dr. Meriam's paper, see page 617.)

*The President.*—Gentlemen, Dr. Meriam's very interesting and practical essay is before you for discussion. I hope you will all speak upon it.

DISCUSSION.

*Dr. S. E. Davenport.*—I remember being particularly enthusiastic over Dr. Meriam a few years ago. He favored us with a few remarks in discussion of a paper which was both interesting and valuable, and those remarks pleased us all so much that at the close of the meeting I was impertinent enough to say to Dr. Meriam that I thought his extemporaneous remarks were even better than his writings. I thought I was paying him a very great compliment. Since hearing his paper to-night, and the previous one from his pen, I feel that I must apologize to him, and say that his writings are quite equal now to his extemporaneous remarks.

It is well known that no matter how barbarous a country may be, if visited by Christian missionaries not only are souls saved, but the practical side of life is looked after by the inhabitants of that country in a much better manner; the people keep cleaner and transact their business with more energy than they did before the missionary's visit.

I look upon Dr. Meriam as our own bright, special missionary. He keeps coming, and every time he comes there is a radiance,—we see the sign of it when he takes the position back of the essayist's desk,—there is a halo about him which does not come altogether from the dinner just eaten, and I feel that he is elevating these New Yorkers,—and we all need it,—he is increasing the dignity of our profession.

His work is certainly arduous. It is freely given,—done entirely for the love of it; and I wish to express my individual obligation to him, and to say that, if left to ourselves, we might possibly continue to make very fair fillings, but at the end of the day we would not feel that our mission had been such a high one after all, as we may if we accept Dr. Meriam's estimate of our work, and his idea of what we can make of our specialty.

*Dr. J. Morgan Howe.*—I feel very much in accord with the sentiments that have been offered by my friend, Dr. Davenport, in the view he takes of the missionary character of the visits that Dr. Meriam makes to us, and the missionary effect that they have on us.

I really think that we are in a sense cleaner, ethically, than we used to be; that we live on a little higher plane than we did at one time, and I look hopefully to the future.

There is very much in what Dr. Meriam has said that concerns us practically. The time is too short to refer to all, but some of the points to which he just referred appear to me to be very suggestive, in that so little has yet been done by dentists to increase the knowledge among dentists of the materials which we use in our practice. We really know very little of the materials which we use; we have, to a great extent, left this matter in the hands of dealers and manufacturers who have served us with what they chose.

It has appeared to me, and it is a suggestion that comes very naturally out of Dr. Meriam's remarks to-night, that societies could be engaged in no better work, through committees and otherwise, than in endeavoring to fix upon the constituents of alloys which would be best calculated to serve our purpose, which would be best worthy of our experimental uses, and cements which may offer opportunities for improvement, if a study of them were entered upon in conjunction with chemists, and with manufacturers, some of whom undoubtedly would be willing to co-operate with societies in such labors.

Very many of the materials might be improved, and some which we do not use might be brought into use, by such efforts as these. The resources of, and relations to, all the learning and all the science of the world that we have at our call and demand are very great; but as yet our demands have been almost nothing. We have not reached up for what the world of science has to offer us, and in this particular direction alone Dr. Meriam's paper contains a great deal of very valuable suggestion, which I hope we may in the future act on from a practical point of view.

*The President.*—Applying the suggestions made by Dr. Meriam's excellent paper to the larger experience of Dr. Lord, I think he is ready to give us some hints that will be of value to us.

*Dr. Lord.*—Mr. President, I think it very doubtful whether I shall be able to say much that will be edifying.

I do not think I know much, and, as I grow older and have more experience, I feel that I know less.

I have often been led to wish that I had spent more of my life in study and in investigation, that I might the better understand the true relation of things and their intrinsic value, and less in actual work with my hands.

No professional man—indeed, this may be said of men in every department of life in this most active and progressive age—can wholly depend upon his experience, however extensive it may have been. Every one should be a student as well as a practical worker, if he would be thoroughly furnished for his work.

It can be said that the practice of the art of dentistry is made up, in a great measure, of most careful and exact manipulations, but there are great principles—the principles of physiology and pathology—which underlie the application of the art, and these must receive all due attention if we would hope to cover the ground as well as we ought to.

The possibilities of our calling, which, as I understand it, is one of the leading thoughts of the paper, are as yet by no means reached or understood—perhaps hardly even entered upon—by the profession at large.

We know altogether too little of the materials and medicaments that we use, particularly those of a compound character.

Take, for example, the alloys we use, and how little we know of their composition, or whether the formulas will give us the very best alloy that can be made. Then, if the formula is all right, how very seldom can we be sure that the different metals had been refined and were pure before they are melted together.

Then, again, if I understand it, the formula may be destroyed, at least in a measure, unless the melting of the metals is done with the greatest care, by the burning away of some portion of the softer metals.

We are altogether too dependent upon the manufacturers of the materials that we use, and this may be said also of our instruments; not that we can manufacture for ourselves, but we should be able to tell what we want, and only employ such persons as will be sure to give us just what we order.

I think I am using, to-day, a better alloy than I ever used before. I recommended it to a friend, who asked me the price, and when I told him three dollars he remarked that the price was high and that he could make an alloy himself much cheaper.

Is there not altogether too great a disposition to use cheap or low-priced materials, and should we not consider that the best is the cheapest? If it required ten dollars an ounce to produce the very best alloy that could be made, we ought not to hesitate to give it.

It has been said, and no doubt with much truth, that there are prepared for our use too many or too great a variety of forms of gold. They are not needed, and are only more or less embarrassing, particularly to the younger men.

As has been said, they are prepared more in the interests of the manufacturers, and are no doubt often sold on account of the promising and extravagant manner in which they are advertised.

We should no more expect or allow the makers and venders of dental goods to tell us what we need and should use than the druggist should tell the physician the kind of remedies and drugs he should use in his practice.

*Dr. S. G. Perry.*—I admire New England; I have a great admiration for that rock, and for that ship which came over. It is said of New England that she was the cradle of liberty. Certainly slavery was wiped off the face of some of the earth through her efforts.

There is a certain ruggedness about the character of her sons that I always admire. Perhaps it comes from tilling her rocky soil, from fighting the Indians in the early days, or from eluding the malaria that was said to infest her valleys. She had always manifested a desire for liberty, and the same spirit is shown in Dr. Meriam's paper to-night. There is an undercurrent of meaning in it that suggests a great deal. There is also a foreshadowing of the individual duty of men who enter a profession, a sort of moral obligation that rests upon any one who enters a liberal profession like ours.

Is life worth living if we cannot feel and manifest an interest in the profession we have chosen? It may be doubtful if it is worth while to struggle through life unless we can have a little of the feeling that, though we work modestly and quietly, our efforts shall yet be a benefit in some way, and that we shall reflect some credit on the profession.

I was profoundly impressed with what Dr. Dwinelle read to-night in reference to Dr. Maynard. What a life was that! What an inspiration it ought to be to every man in this room, to think of the ability of that man and the devotion he showed to his calling! If our point of observation is right, this world is worth living in, and there is something to live for. That which has been greatest and best in this country has, for the most part, had its origin in New England, and I think we are under obligations to our friend for coming down here in his missionary work.

*Dr. C. E. Francis.*—Mr. President, although a New Englander, I do not feel that I can add anything of interest to what has already been said this evening. I believe that not only dentists, but all people, as a rule, are better off for having a broad and generous knowledge of things in general. It is well for dentists to be able to do very many things, but I sometimes doubt if a man can be eminent in any one branch or specialty of science or art and still figure as a "jack-at-all-trades." It is right that dentists should know how to make alloys, instruments, etc., yet it seems to me that operative dentists of the present period can employ their time and talent to better advantage in other ways than in doing these things.

In my early days I melted and prepared my gold plate and solder and shaped my instruments, but do not feel inclined to do so at the present time. I prefer to have manufacturers who make a specialty of such work do these things for me. They can do them better than I can, and this leaves me more time for other duties.

*The President.*—I would call on Dr. Brockway, as a representative from Brooklyn.

*Dr. Brockway.*—Mr. President, there are more worthy members from Brooklyn present than myself, but while I am on my feet I will take occasion to express my gratification at Dr. Meriam's paper, which I have listened to, as I always do to his efforts, with extreme satisfaction. I recognize that the New England spirit, of which this paper is an exponent, is something that goes back of New England itself. That is, it is a spirit of independence, and of seeking after truth and liberty, and I am sure that that spirit cannot fail to be of advantage to any profession which adopts it.

I was impressed somewhat with the remarks of Dr. Lord, that if he had his life to live over again he would devote less time to matters of detail and practice, and give more time to an investigation of the causes of things, and why certain combinations of materials are better adapted for the case in hand than others. You all know the old proverb,—"Happy is the man who understands the causes of things!"

If we could understand the causes of things, we should save ourselves a vast amount of discomfort and anxiety, and would be able to give our patients the benefit of exact knowledge, where usually we are only able to give them the benefit of mere assumptions. I never listen to Dr. Meriam without feeling encouraged and stimulated to better effort; he makes me feel that I am better qualified to cope with the difficulties that I encounter than I would have been, had I not heard him.

*Dr. Farrar.*—When I came here I had no thought that I would be called upon to speak, but as long as I have been, I will say that I have been much interested in the paper, and believe in its teachings, heart and soul. We all, as dentists, work too much like bees who carry their honey to hive, for man to draw out. We should have more independence of thought and of action; we should know where to get the best of things that we need, at reasonable prices, and we should spread that information broadcast. I long to see the time come when dentists will be able to lay aside a little more money out of their hard earnings, and not be obliged to live the life of property bees.

There are no professional men who work harder than dentists, and while they all enjoy the calling, if they are qualified by nature and education to practise it properly, they should enjoy a larger measure of the "sweets," as it were, as a reward for their hard work. Nothing would please me more than to see the spirit shown in this paper carried out by the profession. I mean the spirit of a fight for freedom, and independence from bondage of any kind, and if I see rightly, this spirit is already aroused "all along the line." If this be true, then sooner or later dentists will be placed in circumstances which will enable them to enjoy that which the merchant enjoys from his labors.

*Dr. E. A. Bogue.*—It seems that those heathen nations that were alluded to to-night not only become that much better, as has been mentioned, upon the reception of civilizing and Christianizing truths, but their pecuniary condition, due to careful trading on those same principles, is improved. Hence I think we may take some encouragement to ourselves that the moment we can adopt a higher principle, that moment may be counted upon as improving us pecuniarily, as well as professionally.

Dr. Meriam has certainly struck within the breasts of the members of this Society a responsive chord. It is not necessary to tell him again that we are learning more and more to sympathize with him in the views he has advanced this evening; but as in our younger days we used to take the opposite side of a question simply for the sake of carrying on a discussion, I beg his permission to take the opposite side just now; and the reason is that in making application as one of the members of your executive committee to a gentleman for a paper to be read before this Society, I was met with almost a prompt refusal, on the ground that papers read here would not be published in the *Dental Cosmos.* I undertook to argue the gentleman out of his decision, but was met by the strong argument that the *Dental Cosmos* had attained a position that made it the most widely read of all the dental journals; if he had taken the trouble to get up a good article, he wished it to be read as widely as possible.

I should like Dr. Meriam to answer all these numerous remarks, and should like to have him tell us why this gentleman is not right. The gentleman referred to says he is not yet fully established, he is not fully known in the dental profession. He has taken a great deal of pains with the papers he has already written, and he wishes his reputation to be more widely extended, and for that reason he wishes the *Dental Cosmos* to publish his article. He would be very glad to bring his paper before the New York Odontological Society, and so long as we adopted the *Dental Cosmos* as our mouthpiece he did give us something.

Now comes the question: Are we sufficiently strong to fight our own battles? Are we yet able to take the position as an independent profession, or an independent speciality, if you please, casting aside personal self-interests and laboring for the good of others? Because if we have really, in the moments or hours of relaxation, or even of work, discovered some of the laws of Nature, or discovered some of their applications, it is our privilege as honorable men, as well as beneficent men, to spread that knowledge as widely as possible, so that if there are any truths in it they may be extended to others.

*Mr. President.*—I am sure we would like to hear from Dr. Carr.

*Dr. William Carr.*—Mr. President, I was very much pleased with the essay and can heartily endorse it. We often use drugs and materials without any knowledge of their composition or of their action, relying solely upon the statements of the chemist or the manufacturer. This course often results in disappointment and failure. I think that the time has arrived when a society like the Odontological should be divided into sections, and that special subjects should be assigned to each section for investigation, allowing ample time in which to report. For instance, the subject upon which Dr. Lord has just spoken—"The Alloys"—is of great importance to every practitioner. I do not agree with Dr. Francis that he has no time to investigate alloys. I should be willing to give my evenings for the greater part of a year if I could thereby solve the amalgam problem.

Yesterday a patient who was formerly in Dr. Bronson's care came to me for treatment. Of eleven copper amalgam fillings, four were complete failures, and I believe that the rest of them will preserve the patient's teeth indefinitely. We all know what a painstaking and conscientious operator Dr. Bronson was. Why were four of these fillings failures? Was it Dr. Bronson's fault? In preparing the amalgam, did he overheat it? Did he squeeze out too much mercury, or leave too much in?

If some one would investigate the subject and give us a perfect amalgam, it would prove a great blessing to humanity The price should not enter into the question at all. I will give to-day fifty dollars an ounce for a copper amalgam that will work perfectly. Give me an amalgam that I can rely upon, and that will preserve the teeth of my patients.

Of course, as I am not doing missionary work like my friend Davenport, I get compensation for my services; so if I gave fifty dollars an ounce for the amalgam, I should expect to be compensated accordingly.

*Dr. J. F. P. Hodson.*—I heartily endorse all that Dr. Meriam has said, and I have been very much delighted with his essay. In respect to some things that have been said by others, I should take some exception.

I cannot believe with them that, in a specialty like ours, involving in the vast majority of operations so much of the very refinement of mechanics, and so much of positive manual dexterity in their application, an education in the mechanic arts is so non-essential. On the contrary, I feel that the usual operations of the purely "medical" dentist show him to have wofully mistaken his avocation. I was educated originally by Dr. Westcott, and one of the prerequisites for graduation from that institution was that every man should make a complete set of his own instruments from the crude materials in addition to the usual laboratory training. I gained thereby a knowledge of the manipulation of metal that has stood me in good stead ever since. It gave me a power of resource and strength of foundation that no theoretical education could take the place of in making special instruments; judging instruments and knowing the good from the bad; making all sorts of alloys, and, indeed, all of those things that are of the practically educational; but I think they were only educational, and that is the especial point I wish to make. I cannot find that one can go on and do these things any more than a physician can go on and manufacture his own medicines or a ship's captain make a business of going aloft to furl sail; his time can be employed to infinitely better advantage, but, with his thorough training in every detail, he is master of his surroundings in a very real sense.

*Dr. Horatio C. Meriam.*—I need not speak of the gratification I feel at the cordial reception you have given my address. I wish, however, to disavow for myself the compliments that were so kindly given, and ask you to esteem me as a follower of what I see should be followed. In the results that come the individual may be nothing. He may be lost in the greatness of some cause he has aided.

The position taken by Drs. Carr and Hodson on the educational importance of the study of these questions and the resulting practical value to us will, I am sure, be found important in the future. In the discussion of alloys, or anything made from a known formula, we shall secure those that, by being taught to others, can be perpetuated, and the discussion on their value will be a mine of information for the future pharmacist. As matters are now, the alloy we use and find good may be changed or cheapened, and we change backward to some former formula unknown, or forward to a new one equally unknown. If the discussion of amalgam in this Society some years since had been on alloys of known composition, it would have added much to that which could be taught, and when in that form they can properly be called "professional," because they cannot be controlled by parties outside our specialty.

The price of articles we use has been mentioned; we pay enough to command the best service. In open making, material and skill in making are charged for, but in a Trust or Combination the price is fixed at "what it will bear." I was told not long since that prices on new things were fixed not by the cost of production, but by the price of a similar article, and that the price of articles similar to those patented was fixed so as to cover the price paid for the patent, that the contrast in price might not be seen. So you see the importance of having articles made outside the combination. We know nothing of the real value of secret preparation; the "ten dollar per ounce alloy" may bear no relation to its cost, the price being made by the fiat of its maker.

I was glad to hear Dr. Hodson refer to the knowledge gained from a training in making alloys, and that physicians did not make their own medicine. Probably very few in full practice study the composition of drugs; but no one has a right to hinder another's investigations and claim to be professional, and no pharmacist or instrument maker undertakes to direct what the physician shall use.

I will try now to reply to Dr. Bogue's question. A representative of one of the Combination journals said to me, in substance, "We do not mean to have dentistry united with anything else; we do not mean to have dental things around in drug-stores or in the shops of dealers in scientific and surgical instruments, etc., but to keep it separate, virtually committing our specialty to isolation from all the other arts and sciences of the world." I do not see how they can take any other position and keep up the Combination. If they control a thing, advertise it; if not, keep it out of the journals, though the government in its postal regulations requires a publisher to make oath that the advertising pages of his journal are open to all firms and dealers.

Do we want journals that represent us, or trade? A librarian who was familiar with some of our trade journals said to me that if it were not for the aid given them by dentists, they would have to be thrown around, like Ayer's Almanac. Now, why should not the gentleman who wishes a large circle of readers publish his paper in Ayer's Almanac? It is fully illustrated, well edited, has a large circulation, has a large capital behind it, its future is assured. The answer is that it is not an authority in medical science, but represents Ayer & Co. We wish to build up a journal that will represent all sides and relations of our specialty, and one that in libraries and by exchanges may be looked on as representing it, and which cannot be suspected of owing other allegiance or representing other interests. When we have done this, our friend will find that his paper, by the reference that it would gain from contemporaneous papers and literature, would gain a larger and more valuable circulation than it is possible for it to receive in journals aiding the isolation of our specialty.

*Dr. E. A. Bogue.*—Mr. President, if you will kindly bear with me a little while, I would like to ask Dr. Meriam another question or two.

What argument is to be offered to these gentlemen, some of them graduates in medicine, all graduates in dentistry, and well-known to us as earnest workers,—what arguments are to be offered to them to induce them to publish their articles in a paper of very limited circulation, as they may tell us? How can we induce them to join with us in the fight for professional independence, which will enable us to say "Go here, go there, go yonder for what you want." Take any of the journals that are known as proprietary journals. Dr. Meriam mentions Ayer's Almanac; well, Ayer's Almanac is a good thing for certain purposes. Why should we not publish our proceedings there? Probably it would be more widely read than even the *Dental Cosmos.*

I should, for my part, like an argument to be put into my mouth, which I may lay boldly before these gentlemen, and receive their answers. May I beg Dr. Meriam to give us a reply?

*Dr. Meriam.*—It seems to me that a man strives for what he likes best; that if one really prefers a dollar to professional reputation, there is no argument. This covers a point that perhaps ought to have been in my paper, which is that the spirit of appreciation would probably come if we saw these things around us and in our literature. Many feel they get no thanks whatever for doing original work.

We are not asking, as I understand it now, for more money; we are not asking for more money from the community for ourselves, but we are protesting against the isolation of a specialty of medicine, and the question comes up again, Are these journals tending to produce this isolation of our specialty, or are they aiding to establish its correlation?

*Dr. Remington.*—I would move that the Society show its appreciation to Dr. Meriam by a vote of thanks.

Seconded and carried unanimously.

Adjourned.

S. E. Davenport, D.D.S., M.D.S.
*Editor New York Odontological Society.*

